# Elemental analysis of single ambient aerosol particles using laser-induced breakdown spectroscopy

**DOI:** 10.1038/s41598-022-18349-8

**Published:** 2022-08-29

**Authors:** Paavo Heikkilä, Antti Rostedt, Juha Toivonen, Jorma Keskinen

**Affiliations:** 1 Aerosol Physics Laboratory, Physics Unit, Faculty of Engineering and Natural Sciences, Tampere University, 33100 Tampere, Finland; 2 Photonics Laboratory, Physics Unit, Faculty of Engineering and Natural Sciences, Tampere University, 33100 Tampere, Finland

**Keywords:** Atmospheric science, Atmospheric chemistry, Applied optics, Optical spectroscopy, Characterization and analytical techniques

## Abstract

Analysing the composition of aerosol particles is essential when studying their health effects, sources and atmospheric impacts. In many environments the relevant particles occur in very low concentrations, meaning that their analysis requires efficient single particle techniques. Here we introduce a novel method to analyse the elemental composition of single aerosol particles sampled directly from the aerosol phase using size amplification aided aerosol charging (SAAC), linear electrodynamic quadrupole (LEQ) and laser-induced breakdown spectroscopy. We present results of the charging and focusing efficiencies of the SAAC and of the LEQ, and a proof-of-concept of the analysis method. The proof-of-concept test series was conducted with particle diameters down to 300 nm, sampled directly from the aerosol phase. The method shows unprecedented performance for spectroscopic submicron particle analysis from arbitrarily low concentrations and has exceptional potential for a portable analysis platform for various applications in the field of aerosol research.

## Introduction

Aerosol particles impact human life in a multitude of ways: they participate in cloud dynamics^1^, cause premature mortality^2^, transmit diseases^3^ and impair visibility^4^, for example. Impacts of aerosols are especially difficult to study, when the corresponding phenomena occur at very low particle number concentrations. Such important and timely phenomena include ice nucleation in the atmosphere^5,6^ and the airborne transmission of infectious diseases such as COVID-19^7,8^. In the atmosphere, the concentration of ice-nucleating particles (INPs) is of the order of a few particles/litre^9^. Moreover, the emission from the human respiratory tract is of the order of few particles/ccm^10^. A key factor when studying the types and sources of INPs is their composition^11,12^. Recent studies have also demonstrated the potential of composition analysis methods in identifying pathogens^13,14^. As the particles are scarce in both environments, the composition analysis should be conducted on a single particle level.

Composition of aerosol particles can be investigated utilizing collection and subsequent laboratory analysis, which enables acquiring sophisticated data ranging from elemental analysis up to single particle spatial composition information^15,16^. However, with such analysis, the temporal resolution of the analysis declines and the time delay between collection and analysis may cause measurement artefacts to the results, caused by compounds evaporating and/or condensing on the sample^17,18^.

An established real-time method to measure the composition of single aerosol particles is aerosol mass spectrometry^19^, which enables to acquire detailed information with rapid sampling rates. However, mass spectrometry requires a high vacuum, expensive equipment, and relatively heavy data analysis and calibration procedures^20^. As an alternative, laser-induced spectroscopy methods, such as laser-induced breakdown spectroscopy (LIBS)^21^, have drawn increasing attention during the last decade^22,23^. LIBS does not require vacuum and can be carried out without complicated sample preparation and at a lower cost compared to mass spectroscopy. Airborne particles are exceptionally suitable for LIBS analysis because the matrix is gaseous. Because of the low density of the carrier gas, the matrix effects are significantly lower than with the more traditional solid substrates^24,25^. However, as LIBS analysis relies on plasma emission induced by a highly focused laser pulse, either the aerosol particle concentration has to be very high^26^ or the particles have to be focused^27–29^. With state-of-the art sheath air focusing and timed ablation, sampling rates of tens of particles/minute for single particles are achievable with concentrations above c.a. 500 particles/ccm. However, relatively high pulse energies of above 100 mJ are required to generate a large enough plasma for repeatable particle ablation^29^.

In this paper we present a method to focus the particles into the plasma region in a reproducible manner, utilizing size amplification aided aerosol charging (SAAC) and linear electrodynamic quadrupole (LEQ^30^) focusing. With the approach, we introduce the real time elemental analysis of single aerosol particles from ambient air with no lower concentration limit. As the aerosol focusing is conducted with an electric field, laser pulse energies below 10 mJ are adequate to fully ablate the particles. Lower laser pulse energy lightens the pulse laser requirements and leads into better signal-to-background ratios, as the plasma volume decreases. The method shows exceptional potential for applications where particle concentrations are low, such as research on atmospheric ice nucleation or aerosol particles emitted from human respiratory tract.

## LEQ-LIBS principle and components

**Figure 1:**
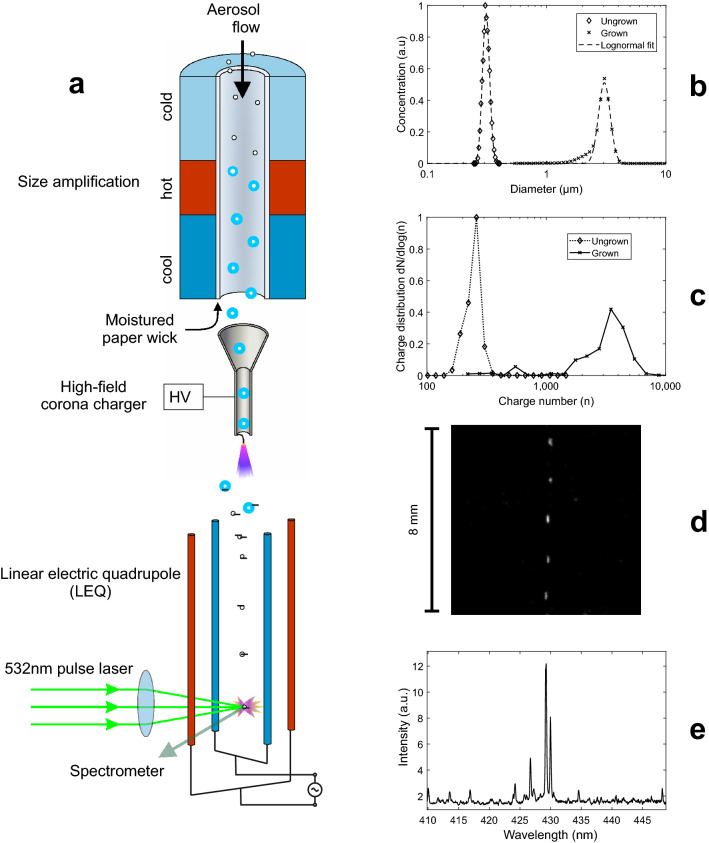
A schematic figure of the LEQ-LIBS analysis. (**a**) The left side illustrates the aerosol flow path through the system. The aerosol first flows through the size amplification, in which condensed water increases the particle size up to ca. 3 µm, as shown in (**b**). After the amplification, the aerosol is charged with a unipolar corona discharge charger, which leads to charging states of several thousand elementary charges/particle (**c**). After the charging, particles are directed into the LEQ, in which an oscillating electric field drives them into the symmetry line, as shown in (**d**). As a single particle drift through the analysis spot, it is detected with a separate 405 nm CW-laser (omitted from the figure), which triggers an Nd:YAG laser, which then turns the particle and the surrounding gas into plasma with a laser pulse having ca. 7 mJ of energy. As the plasma cools down, an elemental emission spectrum is recorded with a spectrometer and an ICCD camera (**e**).

The analysis system is presented schematically in Fig. 1a and in more detail in the Supplementary Fig. 1a,b and the Supplementary Table 1. In SAAC, the sample aerosol first flows through a particle growth tube (Model GTC50, Aerosol Dynamics, Inc.), which consists of a wetted paper wick with three temperature-controlled sections to induce and control particle growing: a cold (10 °C) saturator, a hot (50 °C) heater and a cool (18 °C) moderator. The size amplification occurs in the heater part, as water diffuses from the wick into the aerosol at a faster pace than heat^31^. As shown in Fig. 1b, the particle diameter after the amplification has a median at about 3 µm. After the size amplification, the aerosol flows through a unipolar corona-discharge based aerosol charger, presented in more detail in earlier research^27^. As the particles are size amplified, the powerful electric field in the charger causes them to acquire a high electrical charge^32^ (Figs. 1c and 3). After SAAC, the particles are directed into the LEQ-LIBS chamber through a virtual impactor, which concentrates the large particles into the chamber and omits most of the carrier gas. In the chamber, the particles are dried with a small dry sheath air. As the particles dry and thus decrease in size, their electrical mobility increases rapidly. The increased electrical mobility then enables the quadrupole field to focus the particles into the symmetry axis of the electrodes. Furthermore, the Coulombic repulsion force between the particles causes spacing between them, which ensures single-particle operation.

**Figure 2:**
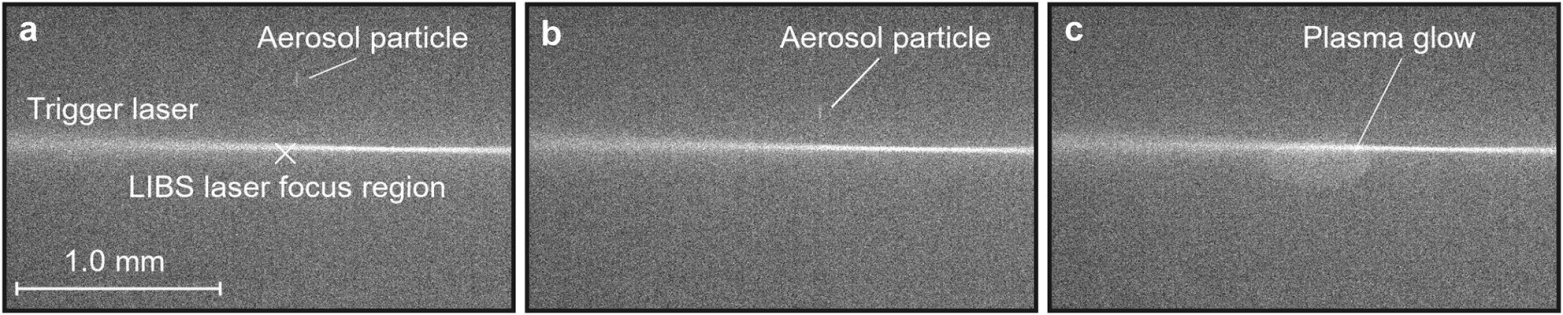
The LEQ-LIBS analysis in action. An aerosol particle with a diameter of 300 nm is approaching the 405 nm trigger laser (**a**, **b**), triggers the LIBS pulse laser and is ablated by it (**c**). A frame between (**b**) and (**c**) is overexposed, but (**c**) shows the actual plasma volume, as it is still slightly glowing after the pulse.

As a particle moves with the airflow along the symmetry axis (Fig. 1d), it crosses with a CW laser beam (405 nm, 100 mW), perpendicularly focused into the axis (Fig. 2a,b). The 405 nm light scattered by the particle is detected by a photomultiplier tube, which instantly triggers a 532 nm Nd:YaG pulse laser with the aid of a DAQ-card. As the particle flow is of the order of 1 mm/s, no additional timing scheme is needed for the triggering. The 532 nm laser pulse, focused into the intersection of the symmetry axis and the trigger laser, turns the particle into plasma, which emits photons at element-specific wavelengths as it cools down (Fig. 2c). The emission spectrum is recorded with a spectrometer and an ICCD-camera. Figure 1e shows an example spectrum from a single tungsten particle. The particles are monitored with 2 CCD-cameras to aid the focusing of the lasers. The triggering and voltage control system is controlled with 2 DAQ-cards and a LabView software.

## Characterization methods

### SAAC charging performance

The charging states reached by the SAAC were measured using monodisperse DEHS-particles generated by the single charged aerosol reference (SCAR). The detailed measurement setup is presented in Supplementary Fig. 2b. First, the SCAR-generated aerosol was divided into a CPC (3756, TSI Inc.) to monitor the total concentration and into the SAAC to charge the aerosol. The temperatures of the saturator, the heater and the moderator were set on 10 °C, 50 °C and 18 °C, respectively. Then, the size-amplified aerosol was charged using a total electric current of 20 µA and a 2 lpm flow rate in the charger. After the charger, the aerosol was dried and introduced into another CPC (3750, TSI Inc.) through a DMA (3085, TSI Inc.). When scanning voltages with the DMA, the number concentration measured by the CPC is directly proportional to the density function of the particle charge $\frac{dN}{dlog\left(n\right)}$, as demonstrated earlier^27^.

As DEHS particles are hydrophobic, isopropanol (IPA) was introduced into the aerosol by driving the aerosol generated by SCAR through a heated IPA container. This procedure caused the DEHS particles to absorb IPA, which is soluble to water. This addition of IPA enabled the size amplification of initially hydrophobic particles in the SAAC^33^.

### LEQ-LIBS performance

The performance of the LEQ-LIBS system was evaluated in terms of hit ratio and analysis speed as a function of aerosol concentration using 300 nm NaCl particles. The measurement setup flowchart is presented in more detail in Supplementary Fig. 2a. In the proof-of-concept phase, the particles were generated using a custom-made bubble generator, which generated bubbles from four 0.5 mm spherical nozzles into a 1-% NaCl water solution. After the generation, the aerosol was introduced into a chamber with a volume of 10 L, which acted as a residence time chamber and a dryer, as dry pressurized air was introduced into it alongside with the sample aerosol. From the chamber, the sample was flown through an AM-241 neutralizer, an impactor with a cut point of about 420 nm and a differential mobility analyser (DMA, 3081A, TSI Inc.), respectively, with a flow rate of 2 lpm. After the DMA, the sample was humidified and divided into a condensation particle counter (CPC, 3776, TSI Inc.) to monitor the concentration and into the SAAC-LEQ-LIBS system for the elemental analysis. The total flowrate into the SAAC was 2 lpm, of which 0.1 lpm was directed into the LEQ through a virtual impactor. When adding the 0.25 lpm dry sheath air into the LEQ, the total flowrate through it was 0.35 lpm. The LIBS spectra were automatically analysed and considered as successful if the signal peak value was 1.5 times the mean of the background value. Additional spectra were analysed from tap water residual particles and Arizona test dust particles.

### LEQ focusing efficiency

Particle focusing in the LEQ was evaluated numerically. Electrodynamic balance (EDB) systems have been used in aerosol research for decades, and their performance is also evaluated and documented mathematically^34,35^. In the following some of the earlier work is applied to the LEQ geometry to provide tools for its particle focusing performance. In the absence of external forces, the force equation for a charged particle in an oscillating electric field is, as Newton’s 2nd states:

1 $$-\frac{3\pi \eta {d}_{p}}{{C}_{c}}\frac{d{\varvec{r}}}{dt}+q{E}_{AC}\left({\varvec{r}}\right)\mathrm{cos}\left(\omega t\right)- m{\varvec{g}}=m\frac{{d}^{2}{\varvec{r}}}{d{t}^{2}},$$

where ${d}_{p}$, $m$, $q$ and ${\varvec{r}}$ are the diameter, mass, electrical charge and the position of the particle, respectively, $\eta$ is the viscosity of the carrier gas, $\omega$ is the angular frequency of the oscillating electric field and ${C}_{c}$ is the Cunningham’s correction factor for small particles. As the particles are flowing parallel to gravity in the LEQ, the focusing is only considered in its perpendicular dimensions. Gravity is thus omitted from the equation, leaving

2 $$m\frac{{d}^{2}{\varvec{r}}}{d{t}^{2}}+\frac{3\pi \eta {d}_{p}}{{C}_{c}}\frac{d{\varvec{r}}}{dt}-q{E}_{AC}\left({\varvec{r}}\right)\mathrm{cos}\left(\omega t\right)=0.$$

The above Eq. (2) is often expressed with dimensionless variables $Z=\frac{z}{{z}_{0}}$ and $\tau =\frac{\omega t}{2}$, which describe the position along an axis of interest and the number of concurred oscillations in the electric field, respectively^36^. The expression includes parameters $\delta =\frac{36\eta }{{C}_{c}\omega \rho {d}_{p}^{2}}$ and $\beta =\frac{24{C}_{1}q{V}_{ac}}{{\omega }^{2}{z}_{0}^{2}\rho \pi {d}_{p}^{3}}$ which describe the drag force and the electric force exerted to the particle, respectively. In the parametrization, ρ is the density of the particle, ${z}_{0}$ is the distance from the edge of an electrode into the symmetry axis and ${C}_{1}$ is a geometric constant describing the slope of the oscillating electric field^27^. The parametrization leads to a dimensionless equation

3 $$\frac{{d}^{2}Z}{d\tau }+\delta \frac{dZ}{d\tau }+2\beta Zcos\left(2\tau \right)=0,$$

from which stability areas with respect to $\delta$ and $\beta$ can be numerically calculated^36^. If the position of the particle along the Z-axis is solved (MATLAB, The MathWorks Inc.) and its average plotted as a function of the dimensionless time $\tau$, one can easily see that it follows an exponential function with a negative exponent (Supplementary Fig. 3):

4 $$Z\left(\tau \right)={Z}_{0}{e}^{-\tau /{\tau }_{ef}}.$$

In Eq. (4), ${Z}_{0}$ is the initial position of the particle and ${\tau }_{ef}$ is a factor describing the time it takes for a particle to drift into the focus spot, i.e. relaxation time in an electrodynamic balance. By solving the differential Eq. (3) with a multitude of different parameter δ and β values and comparing the results with Eq. (4), the relaxation time can be found to be (Supplementary Fig. 4):

5 $${\tau }_{ef}=\left(1+\frac{4}{{\delta }^{2}}\right)\left(\frac{{\delta }^{3}}{{2\beta }^{2}}\right).$$

If written in the context of Eq. (2), including dimensions, (4) and (5) can be described as

6 $$r\left(t\right)={r}_{0}{e}^{-t/{\tau }_{ef}^{,}},$$

and

7 $${\tau }_{ef}^{,}=\left(1+\frac{{\rho }^{2}{C}_{c}^{2}{d}_{p}^{4}{\omega }^{2}}{324{\eta }^{2}}\right)\frac{81{\pi }^{2}{\eta }^{3}{z}_{0}^{4}}{\rho {C}_{1}^{2}{V}_{ac}^{2}{q}^{2}{C}_{c}^{3}}=\left(1+{m}^{2}{B}^{2}{\omega }^{2}\right)\frac{{z}_{0}^{4}}{2mB{{Z}^{2}C}_{1}^{2}{V}_{ac}^{2}}.$$

In Eq. (7), $B$ and $Z$ are the mechanical and electrical mobilities of an aerosol particle, respectively. The Eqs. (4)–(7) can be used to evaluate the focusing efficiency of an EDB in general. This can be helpful in designing a focusing system on which the charging states of the particles is not initially high.

**Figure 3:**
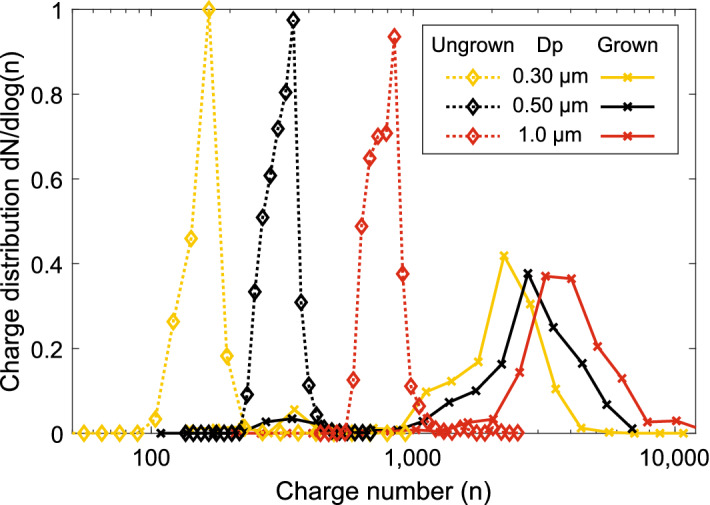
Charge states achieved with the SAAC. The dotted lines present charging states for dry particles, and the solid lines for the size amplified particles. The legend entries present the dry particle diameter. As can be seen from the figure, the dry size has some effect on the final charge of the grown particles. All the curves have been normalized due to their area in the logarithmic scale.

Equations (6) and (7) were used to estimate the time that is needed for the particles to drift within a 20 µm radius of the focus line. This radius can be assumed as a threshold for a successful LIBS analysis in the LEQ, as demonstrated in earlier research with similar optical setup and LIBS pulse energy^37^. Results of the calculated times are shown in Fig. 5. In the calculations, ${C}_{1}=0.77$ was determined from electric field simulations (COMSOL Multiphysics, COMSOL Inc.) inside the LEQ with the used electrode configuration, presented in supplementary Fig. 1b. The charging states were set to 2000, 4000 and 8000, which are in the modes of grown particles’ charging states of Fig. 3, the amplitude AC-voltage was set to 2 kV, frequency to 1 kHz and particle sizes from 100 nm to 10 µm with unity as density. Also, as a comparison, similar calculations are shown for particles charged without the size amplification, but instead with charging states following the equation for ungrown particles with the same aerosol charger^27^.

## Characterization results

### Size amplification aided aerosol charger (SAAC)

The final charging states of the particles charged with SAAC are shown in Fig. 3. As a comparison, the charge distributions for ungrown particles charged with the same corona charger are also shown. The charge distributions have been normalized due to their area in the logarithmic scale. As can be seen from the figure, the size amplified particles reach a similar charging state of a few thousand elementary charges/particle, regardless of their initial size, and the ungrown particles’ charge distributions are more separate. When processed as log-normal, mean geometric charge values of 2300, 2900 and 3700 were fit to the distributions for initial sizes of 0.3 µm, 0.5 µm and 1.0 µm, respectively. A small difference in the final size of the grown particles might cause the slight difference between the final charging states. However, the smaller particles still have a larger electrical mobility due to their smaller diameter and are thus easier to focus in the LEQ.

The median diameter of the particles after the water condensation is found to be ca. 3 µm, as shown in Fig. 1b. The final charge is slightly larger when compared with the charging states without the size amplification at the same size^27^. This is likely due to the different polarity of the charger, as negative ions used in this study have a larger electrical mobility than positive ones.

### LEQ-LIBS proof-of-concept and performance

**Figure 4:**
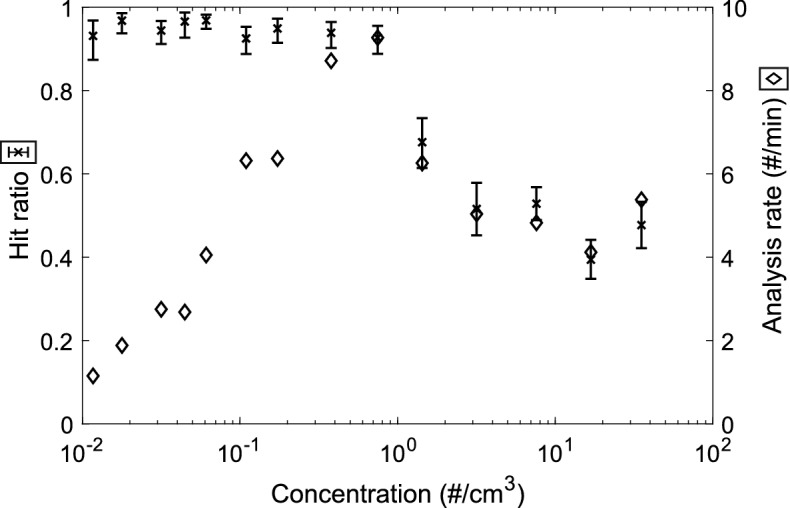
Hit ratio and analysis rate results for 300 nm NaCl particles. The hit ratio (successful emission spectra/laser shots) and analysis rate are presented as a function of ambient particle concentration. As can be seen, the hit ratio is above 90% for concentrations < 1 particle/ccm, which is also the optimal concentration considering the analysis rate. Above that, the hit ratio and analysis rate decrease, which is due to electric interference between the particles in the LEQ. The hit ratio error bars are 95% confidence intervals calculated with the Clopper–Pearson method assuming a binomial distribution^38^.

The results from the LEQ-LIBS performance analysis are shown in Fig. 4. As can be seen from the figure, the hit ratio for ambient concentrations below 1 particle/ccm is well above 90%, and the analysis speed has an optimum at around 1 particle/ccm and is of the order of 10 particles/min. With higher concentrations, the particles experience electrical interference between each other and cause false triggers, which leads into decrease in the hit ratio and analysis rate. Thus, with such concentrations, the aerosol should be diluted before analysis. These results act as a proof-of-concept for the analysis method.

The analysis rate at about below 0.1 particle/ccm is restricted by the small particle concentration itself and between 0.1 and 1 particles/ccm by the LIBS laser pulses: the plasma formation causes a pressure wave into the surrounding gas, which drives the nearby particles away from the focus line, thus requiring some settling time before the next pulse (Supplementary Videos 2, and 4). However, the analysis rate may be improved with more sophisticated temporal flow pattern, i.e., increasing the flow rate temporarily after every pulse or with a faster sheath flow. This could bring the particles unaffected by the previous pulse closer to the analysis spot faster, letting the analysis flow more rapidly.

**Figure 5:**
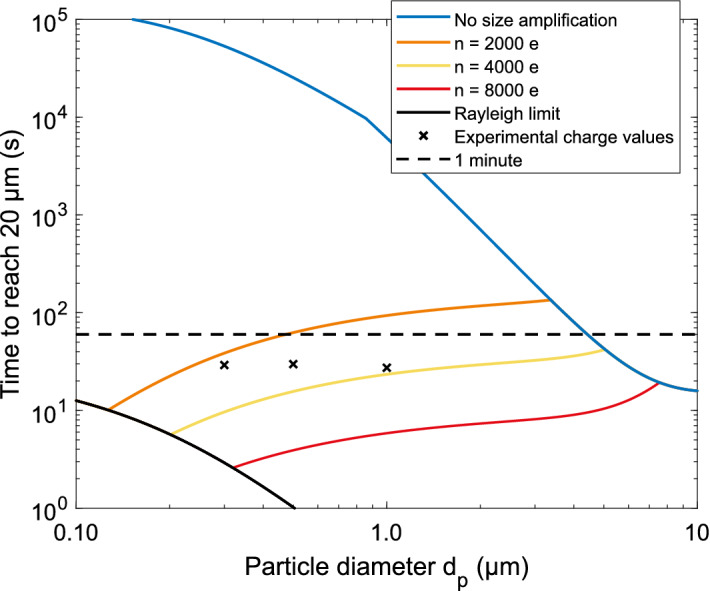
Drifting times toward the focus spot. A radius of 20 µm from the focus line was used as a limit that enables the analysis to succeed, as presented in earlier work^37^. The blue line represents the charge values obtained without the size amplification^27^ and the black crosses the experimental charge values from the SAAC analysis. As can be seen from the figure, particles below a few µm in diameter must be size amplified before charging to reach a reasonable relaxation time.

A radius of 20 µm from the focus line is assumed to be the threshold for a successful LIBS analysis, as experimentally demonstrated in earlier research with a similar optical setup and pulse laser energy^37^. Figure 5 presents results from numerical simulations of the time it takes for a particle to reach the radius with the used electrode setup and several different charging states. When interpreting Fig. 5, it may well be assumed that to be able to efficiently analyse submicron ambient particles with LEQ focusing, the particles must be size amplified before the charging process. However, particles with a diameter greater than ca. 4 µm are charged with respect to their primary size, as the size after the “amplification” would be below that. According to the simulation, the charge these large particles acquire without amplification is high enough for the analysis. The maximum charge limit, namely Rayleigh limit is also presented in the figure for water droplets. As a particle evaporates, it might cross the Rayleigh limit and lose some of its charge via Coulombic fission with a negligible decrease in mass^39^. As can be seen from the figure, the Rayleigh limit ultimately prevents reaching arbitrarily low relaxation times for the smallest particles.

**Figure 6:**
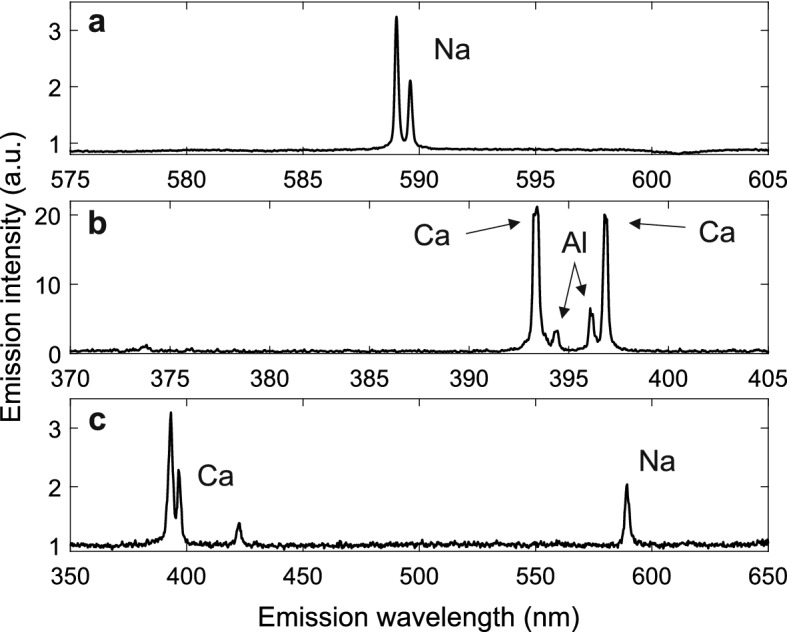
Example spectra from the LIBS analysis. The spectra are divided by a mean of 10 background spectra measured from the carrier gas. The upmost spectrum (**a**) includes the averaged spectra of the hit rate analysis, a total of 3222 laser hits from 300 nm NaCl particles. The middle spectrum (**b**) is from a single supermicron-sized Arizona test dust particle and the last (**c**) from a single residual particle of tap water, also above 1 µm in diameter.

In addition to the hit ratio and focusing performance analysis, example spectra from Arizona test dust and tap water residual particles were analysed. The spectra can be seen in Fig. 6, which contains the average successful spectrum of the performance analysis test series (a), an example spectrum from a single Arizona test dust particle (b) and an example spectrum from a single tap water residual particle (b). The particle diameters corresponding to the spectra were 300 nm (Fig. 6a) and ca. 1–3 µm (Fig. 6b,c).

## Conclusions

A novel method to efficiently control initially neutral submicron aerosol particles in an electrodynamic field to conduct laser-induced breakdown spectroscopy was introduced. LIBS analysis of ambient submicron particles from such low concentrations has not been accomplished in earlier research. The performance of the method was presented on a proof-of-concept level with 300 nm NaCl particles, and LIBS hit ratios of above 90% were achieved for concentrations of under 1 particle/ccm. The optimal particle concentration was found to be of the order of 1 particle/ccm, for which the analysis rate was ca. 10 particles/min. Also, performances of the separate parts of the method were evaluated. The charging states reached with the size amplification aided aerosol charger (SAAC) system were shown to be of about a few thousand elementary charges, which was shown to be enough—and necessary—for efficient focusing of submicron particles in the electrodynamic quadrupole system.

The next major step of development is to conduct measurements of initially unknown aerosols, which requires a wideband emission spectroscopy instrument, such as an echelle- or a multi-channel spectroscope. This is because with a narrow-band emission spectroscope only one or two elements can be monitored at a time, and it is unlikely that the single particles contain just that element. Furthermore, analysing the proportional shares between different elements in single particles is crucial when classifying them. As the test series was conducted with initially neutral aerosol particles sampled from the aerosol phase, the analysis method is expected to work for unknown aerosols as well.

With the SAAC, consistently high charging states were attained. The platform enables the use of any electrodynamic balance for ambient, initially neutral particles without the need for droplet generation and charging systems. Most important development area in SAAC would be to decrease the electric particle losses in the charger, as they were found to be above 70%. Optimising the flow path in the charger, such as adding a sheath flow from the outer tube would prevent the particles from drifting towards the outer walls. Should the particle losses decrease, the analysis rate in the LEQ-LIBS would be higher with a lower initial aerosol concentration.

The analysis rate, which in the current state has a maximum at 10 particles/minute, is also a field of further development. In this study, the most limiting factor was the shockwave caused by the LIBS laser pulse, which drives the nearby particles away from the focus. The effect of the shockwave could be limited by increasing the flow rate in the chamber momentarily after each pulse or by striving towards smaller pulse laser energies by lowering the wavelength, which would possibly lead to a smaller shockwave in the air.

The numerical method to calculate the relaxation time in the LEQ, or an electrodynamic balance in general, may be considered a practical tool to estimate the focusing efficiency for initially uncharged aerosols. The focus in earlier research has mostly been in highly charged droplets^36^, with only few exceptions^40^. However, the simulations have been only partially experimentally verified with measurements^27^.

In summary, the method shows great performance for the online elemental analysis of aerosol particles on a single particle level from arbitrarily low concentrations. Such aerosol environments occur, for example, in atmospheric ice nucleation studies and human respiratory particle studies. Also, the capability to analyse from low concentrations enables the size-segregation of the aerosol population before the analysis without losing all the resolution power. As the used laser pulse energies were below 10 mJ, the method also shows great portability potential.

## Supplementary Information

Supplementary Information.

Supplementary Video 1.

Supplementary Video 2.

Supplementary Video 3.

Supplementary Video 4.

## Data Availability

The datasets and simulation scripts generated during and/or analysed during the current study are available from the corresponding author on reasonable request.
