# Author Correction: Elemental analysis of single ambient aerosol particles using laser-induced breakdown spectroscopy

**DOI:** 10.1038/s41598-024-58193-6

**Published:** 2024-03-29

**Authors:** Paavo Heikkilä, Antti Rostedt, Juha Toivonen, Jorma Keskinen

**Affiliations:** 1 Aerosol Physics Laboratory, Physics Unit, Faculty of Engineering and Natural Sciences, Tampere University, 33100 Tampere, Finland; 2 Photonics Laboratory, Physics Unit, Faculty of Engineering and Natural Sciences, Tampere University, 33100 Tampere, Finland

Correction to: *Scientific Reports* 10.1038/s41598-022-18349-8, published online 29 August 2022

The original version of this Article contained errors. The particle size in Figures 3 and 5 was incorrectly calculated. This slightly affected the particle charge. The corrected particle sizes are 400 nm, 600 nm and 1100 nm and the corrected particle charge distributions are shown in the figures.

The original Figure 3 and 5 and accompanying legends appear below.

**Figure 3:**
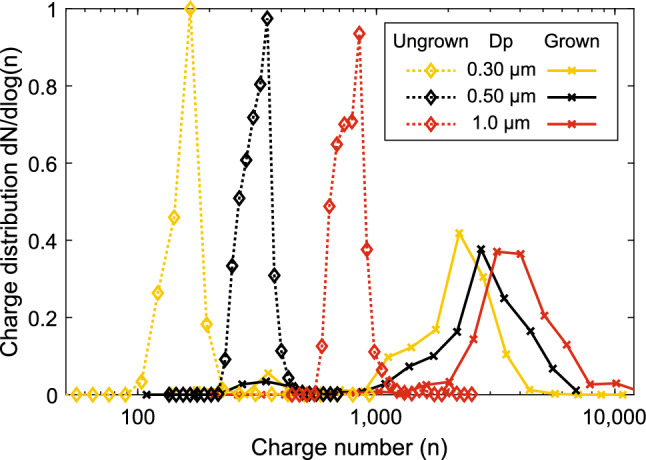
Charge states achieved with the SAAC. The dotted lines present charging states for dry particles, and the solid lines for the size amplified particles. The legend entries present the dry particle diameter. As can be seen from the figure, the dry size has some effect on the final charge of the grown particles. All the curves have been normalized due to their area in the logarithmic scale.

**Figure 5:**
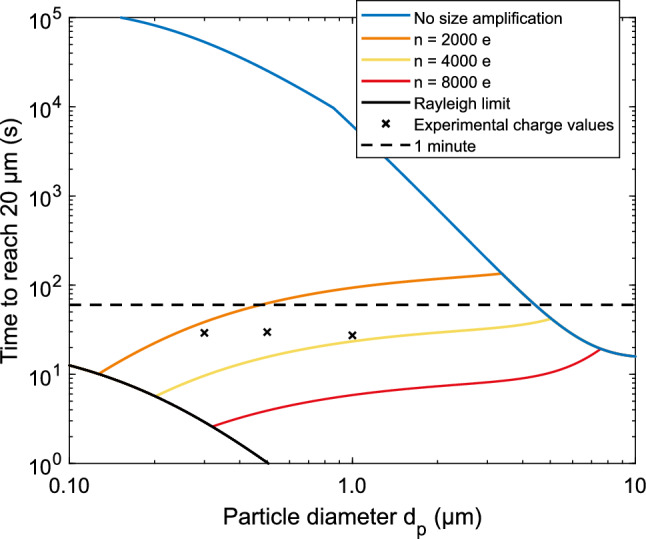
Drifting times toward the focus spot. A radius of 20 µm from the focus line was used as a limit that enables the analysis to succeed, as presented in earlier work^37^. The blue line represents the charge values obtained without the size amplification^27^ and the black crosses the experimental charge values from the SAAC analysis. As can be seen from the figure, particles below a few µm in diameter must be size amplified before charging to reach a reasonable relaxation time.

As the result, in the Characterization results, under the subheading ‘Size amplification aided aerosol charger (SAAC)’,

“When processed as log-normal, mean geometric charge values of 2300, 2900 and 3700 were fit to the distributions for initial sizes of 0.3 µm, 0.5 µm and 1.0 µm, respectively.”

now reads:

“When processed as log-normal, mean geometric charge values of 3600, 4000 and 4400 were fit to the distributions for initial sizes of 0.4 µm, 0.6 µm and 1.1 µm, respectively.”

The original Article has been corrected.

