# Supplementary material for: Elemental analysis of single ambient aerosol particles using laser-induced breakdown spectroscopy
Source: Sci Rep. 2022 Aug 29;12:14657. doi: 10.1038/s41598-022-18349-8 (PMC9421112; doi:10.1038/s41598-022-18349-8)
Supplement: Supplementary file 1 — Supplementary Information. [file 41598_2022_18349_MOESM1_ESM.docx]

Supplementary information for:

Elemental analysis of single ambient aerosol particles using laser-induced breakdown spectroscopy

Paavo Heikkilä, Antti Rostedt, Juha Toivonen, Jorma Keskinen

**Description of the supplementary videos:**

In all the videos, the particles are 300 nm in diameter and the particle material is NaCl.

Video 1 presents the LEQ focusing with aerosol concentrations below 1 particles/ccm and the interference between particles when the concentration is too large.

Video 2 presents the LEQ-LIBS analysis in action with a concentration of about 0.1 particles/ccm.

Video 3 is a slow motion video of the analysis of a single particle.

Video 4 presents the LEQ-LIBS analysis, when the particle concentration is too large. The shockwave generated by the laser pulse can be clearly seen in the video.

**
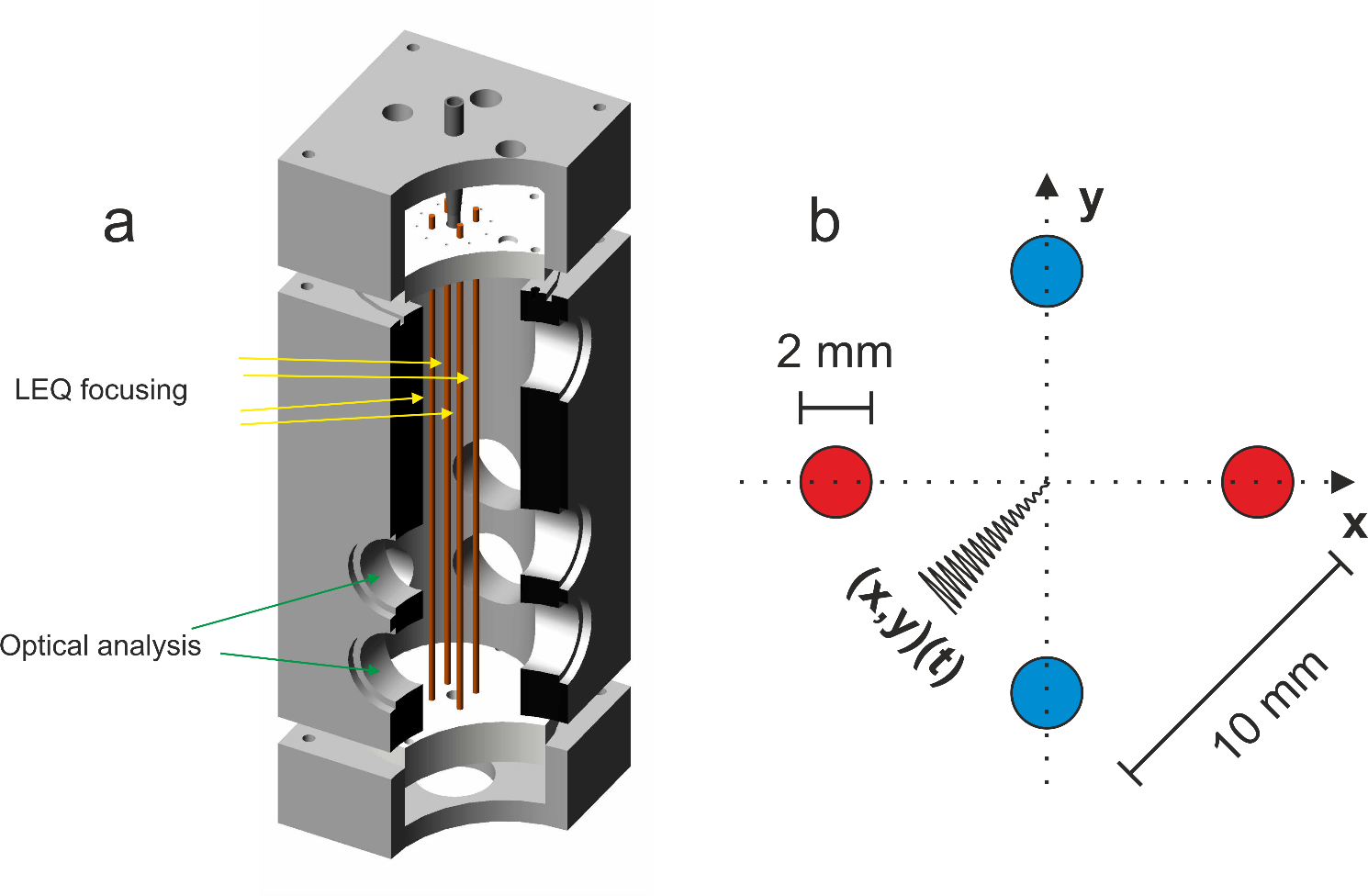
**

Supplementary Figure 1: a CAD-drawing of the LEQ chamber (a) and a schematic figure of the electrodes (b). The highly charged aerosol is directed into the chamber from the inlet on the top, and the copper electrodes carry out the focusing. The monitoring and optical analysis is conducted through the 25.4 mm threaded channels, which contain either lenses or simple windows. The distance between two adjacent electrodes is 10 mm measured from the midpoints.

|  | **Manufacturer and model** | **Unit** | **Quantity** |
| --- | --- | --- | --- |
| **Voltage generator** | National instruments: LabVew,  USB-6363 DAQ-card | Frequency | 1000 Hz |
| **Voltage amplifiers** | Trek: Model 2220 | AC-amplitude | 2 kV |
| **Laser** | Quantel:  Q-smart 100 | Pulse wavelength  Pulse energy | 532 nm  7 mJ |
| **Spectrometer** | Andor: Kymera 328i | Aperture slit width | 25 µm |
| **Detector** | Andor Technology Plc.:  DH340T-18U-E3 | Delay  Collection time | 1 µs  10 µs |
| **Laser focusing and Emission collimation lenses** | Thorlabs Inc:  UV fused silica | Focal length  Diameter | 50 mm  25.4 mm |

Supplementary Table 1: Detailed information about the devices and physical parameters used in the analysis.


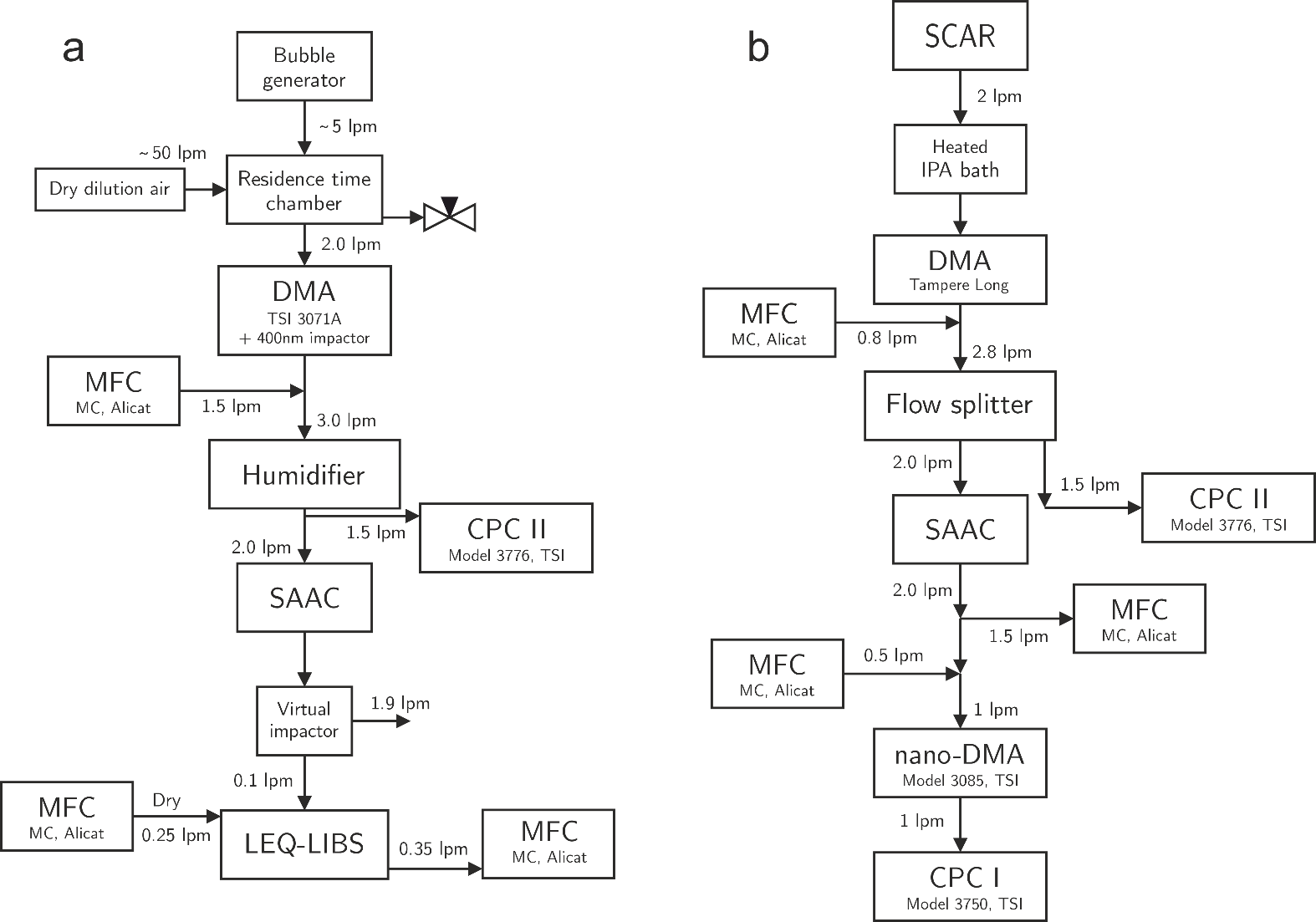


Supplementary Figure 2: The measurement setups in the proof-of-concept measurement (a) and in the charge distribution measurement (b).


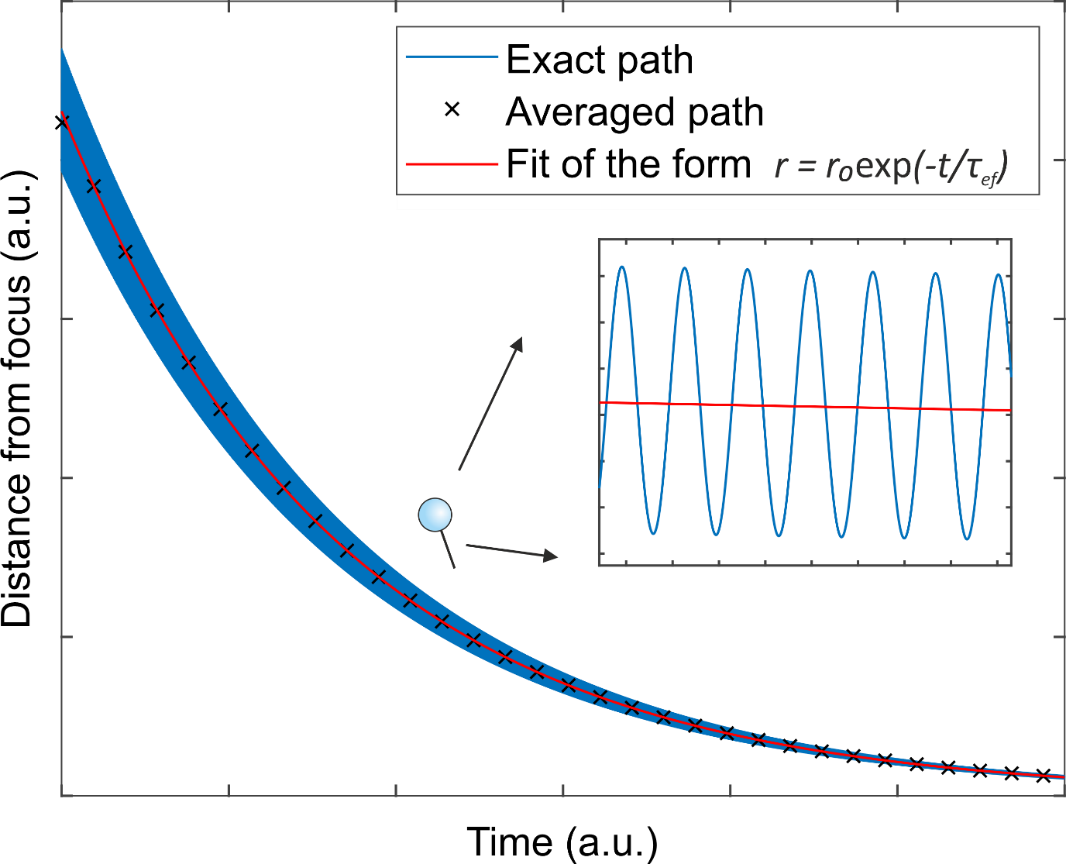


**
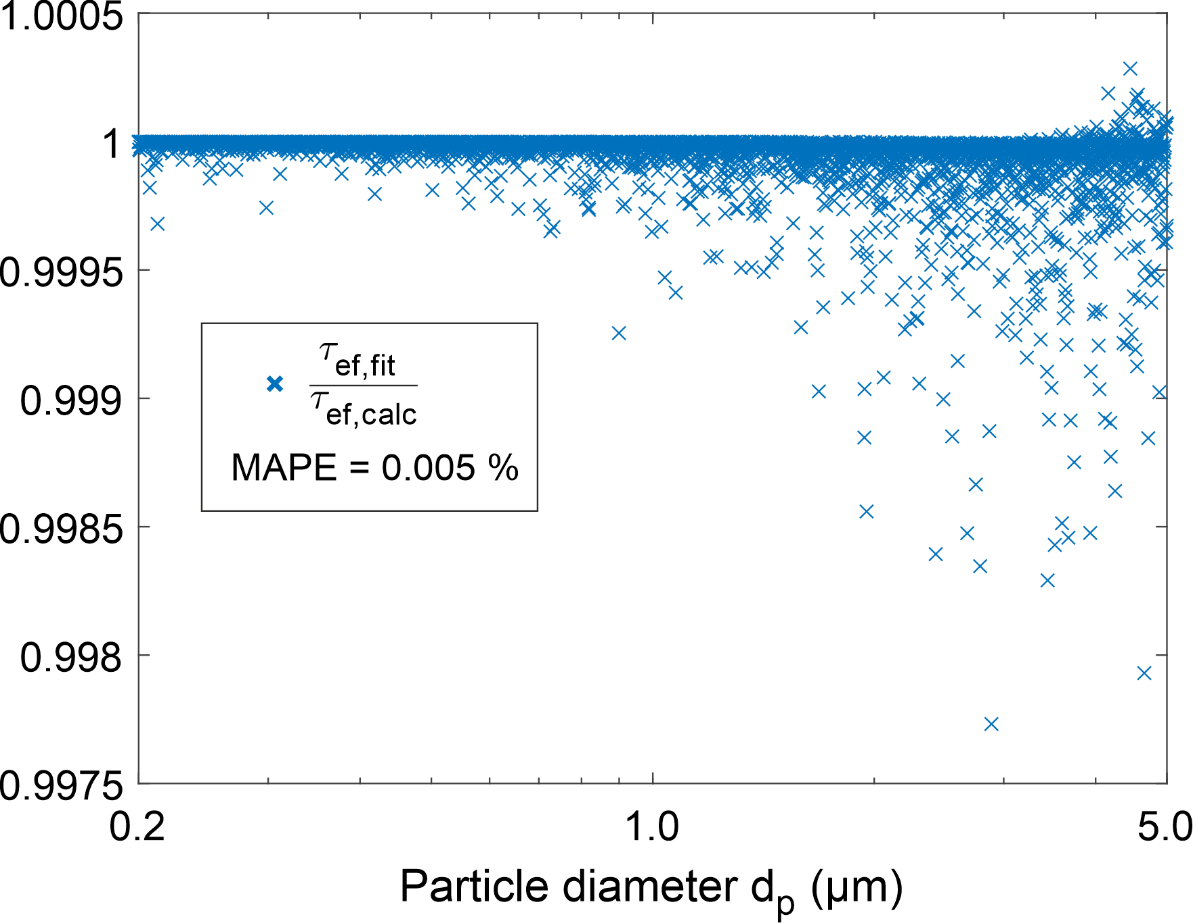
**

Supplementary Figure 4: An example simulation series of the relaxation time. The y-axis contains the quotient when dividing a fitted relaxation time value (the red line in Supplementary Figure 4) with the predicted value from equation (5) or (7). The agreement is excellent, meaning that the equations have great prediction power of the relaxation time in an EDB, according to the simulation.

Supplementary Figure 3: An example simulation of the flow path of a particle along an axis of interest inside an EDB. As can be seen from the figure, the averaged path follows an exponential function defined by the equation in the legend.
